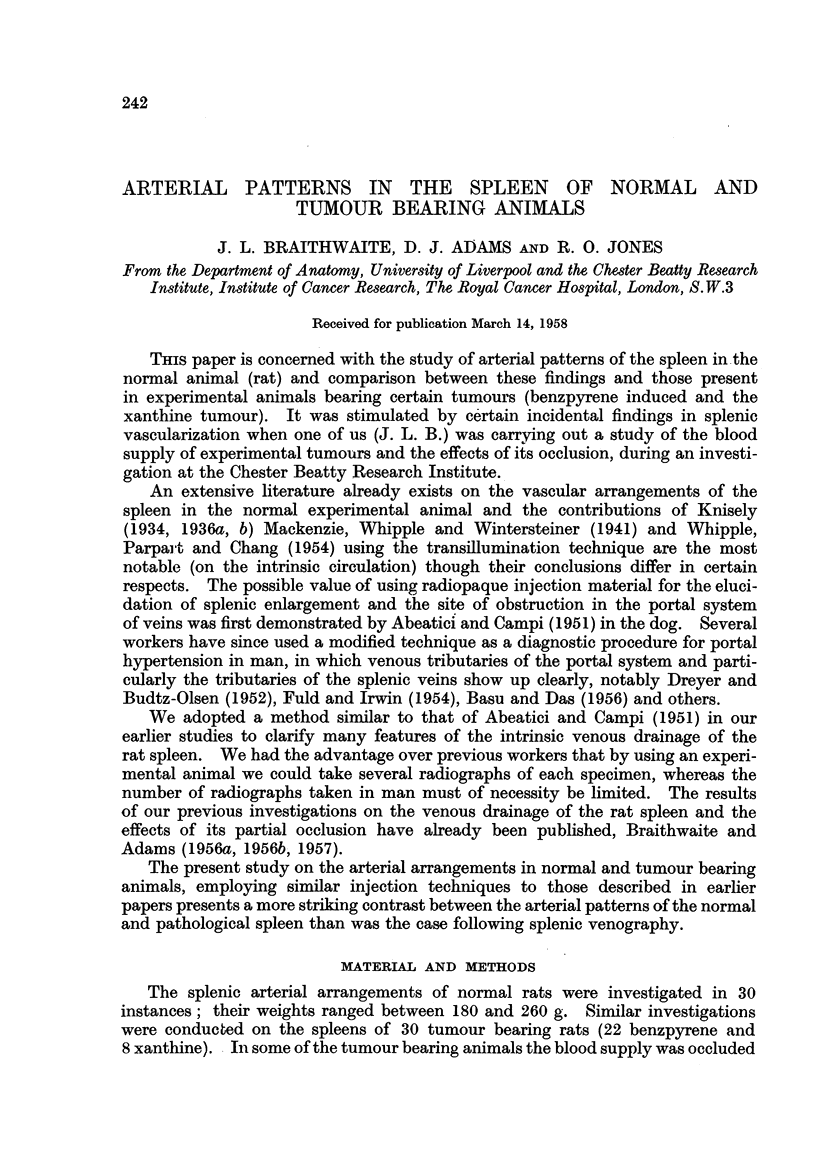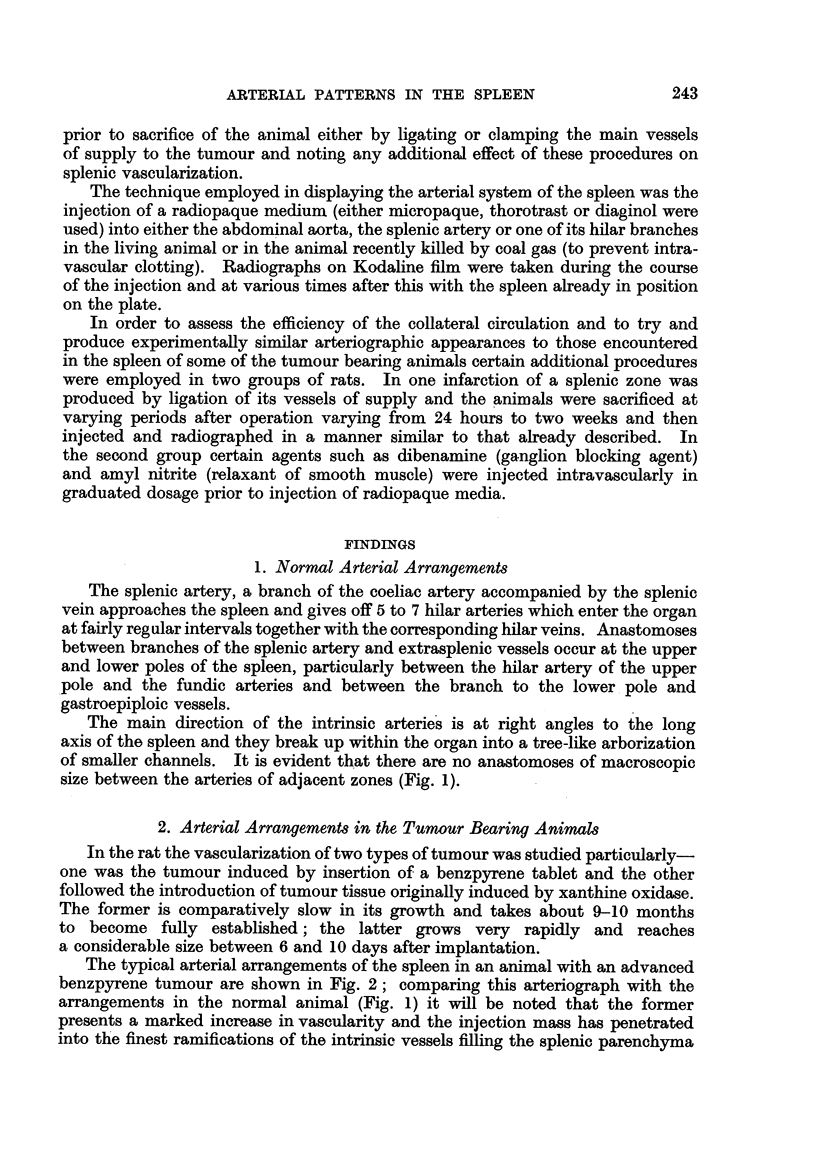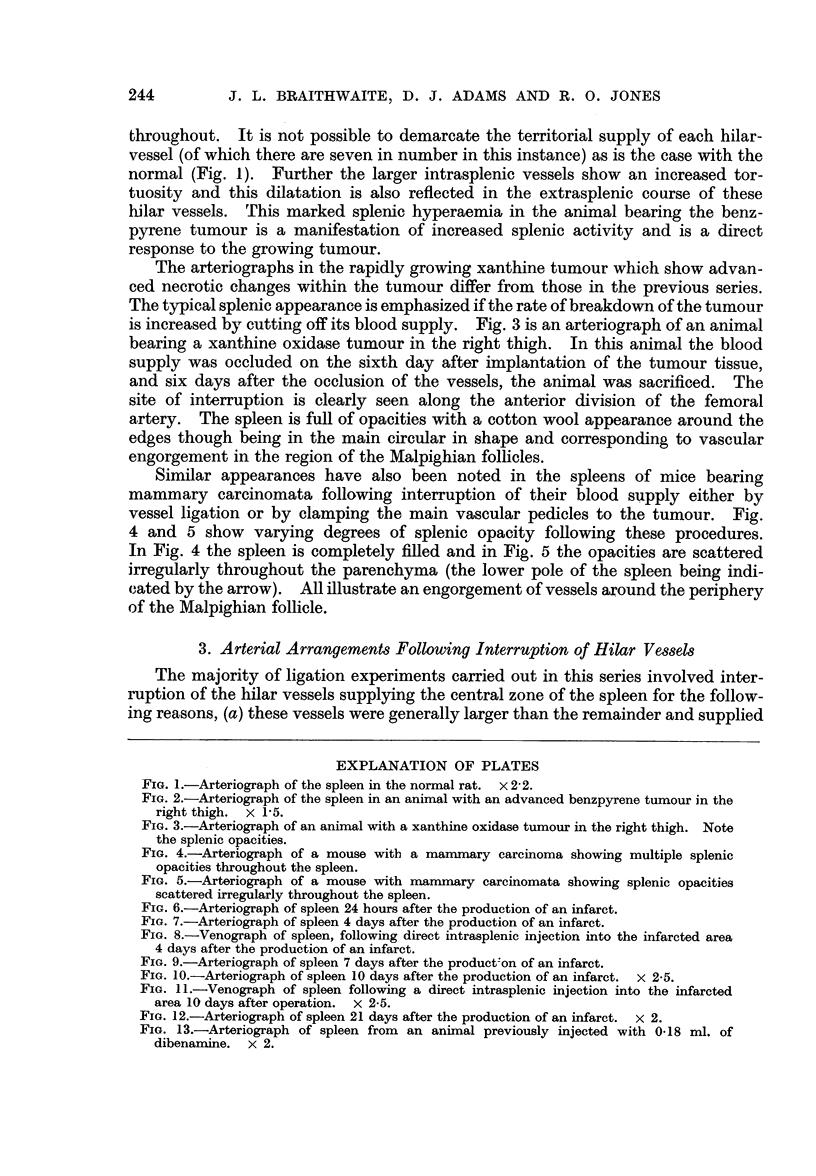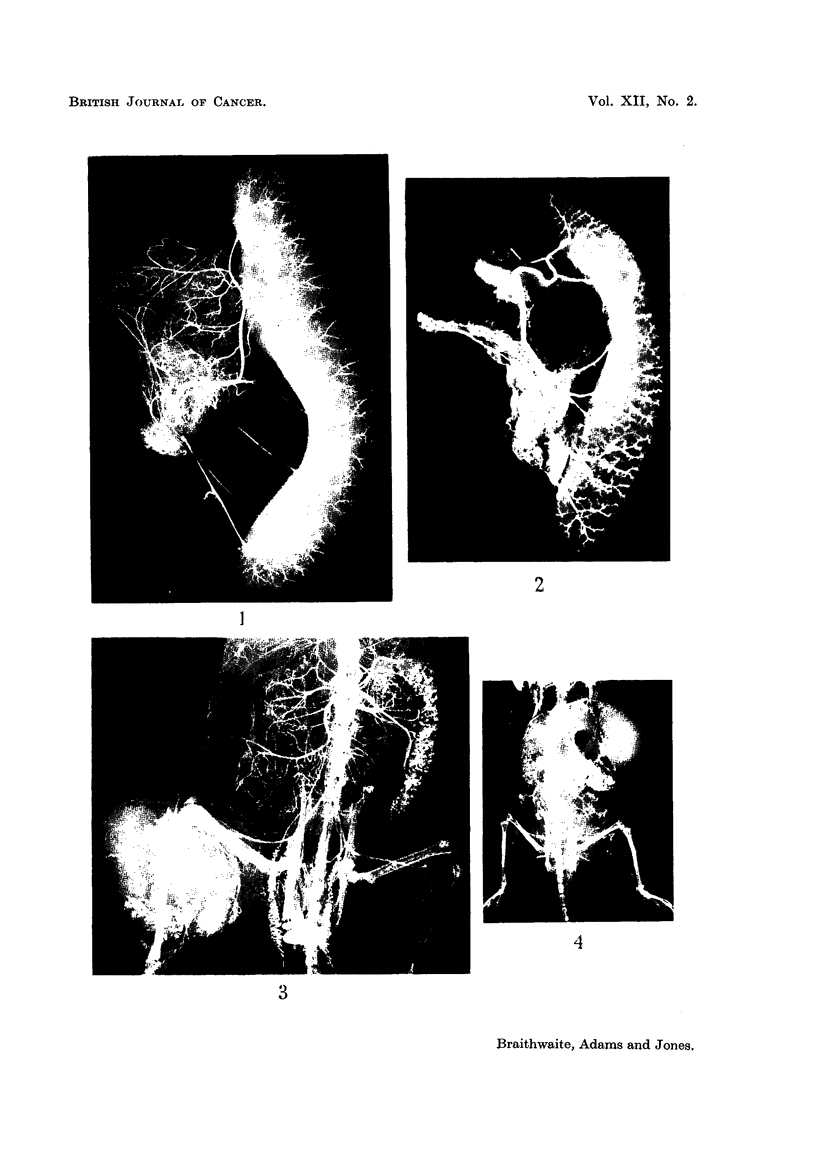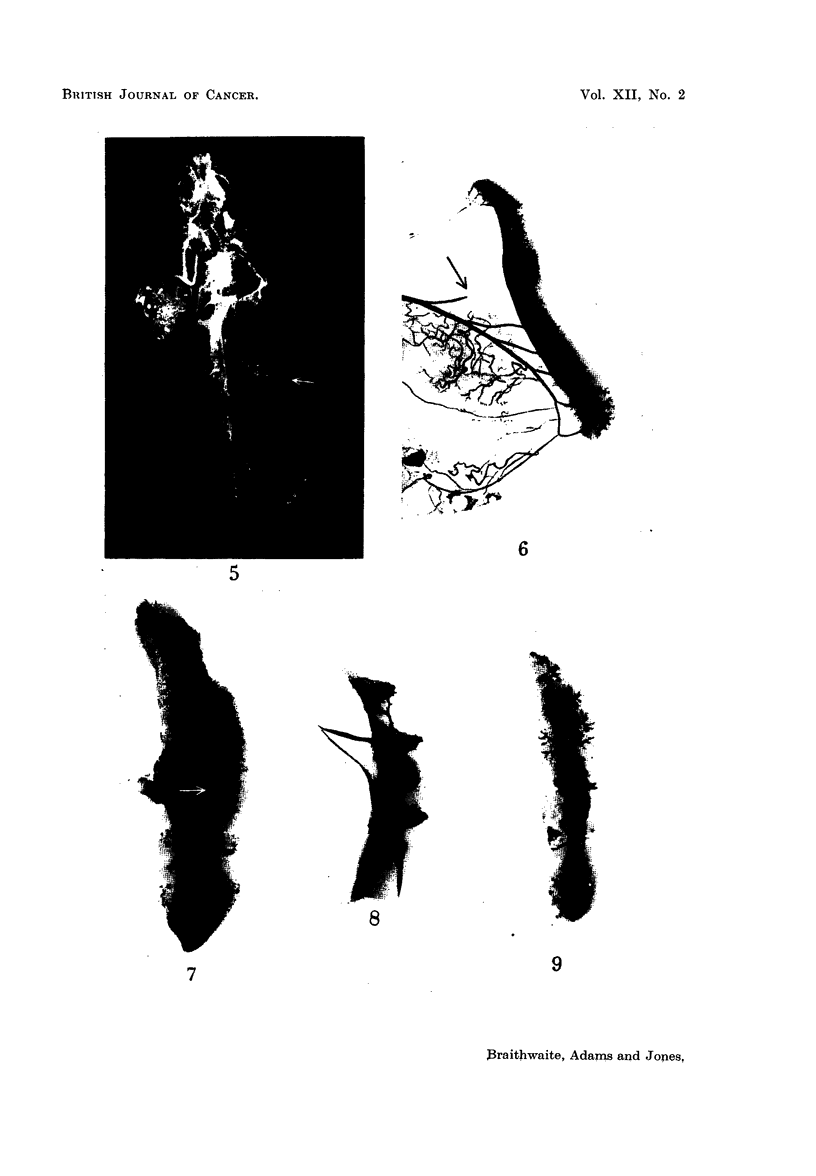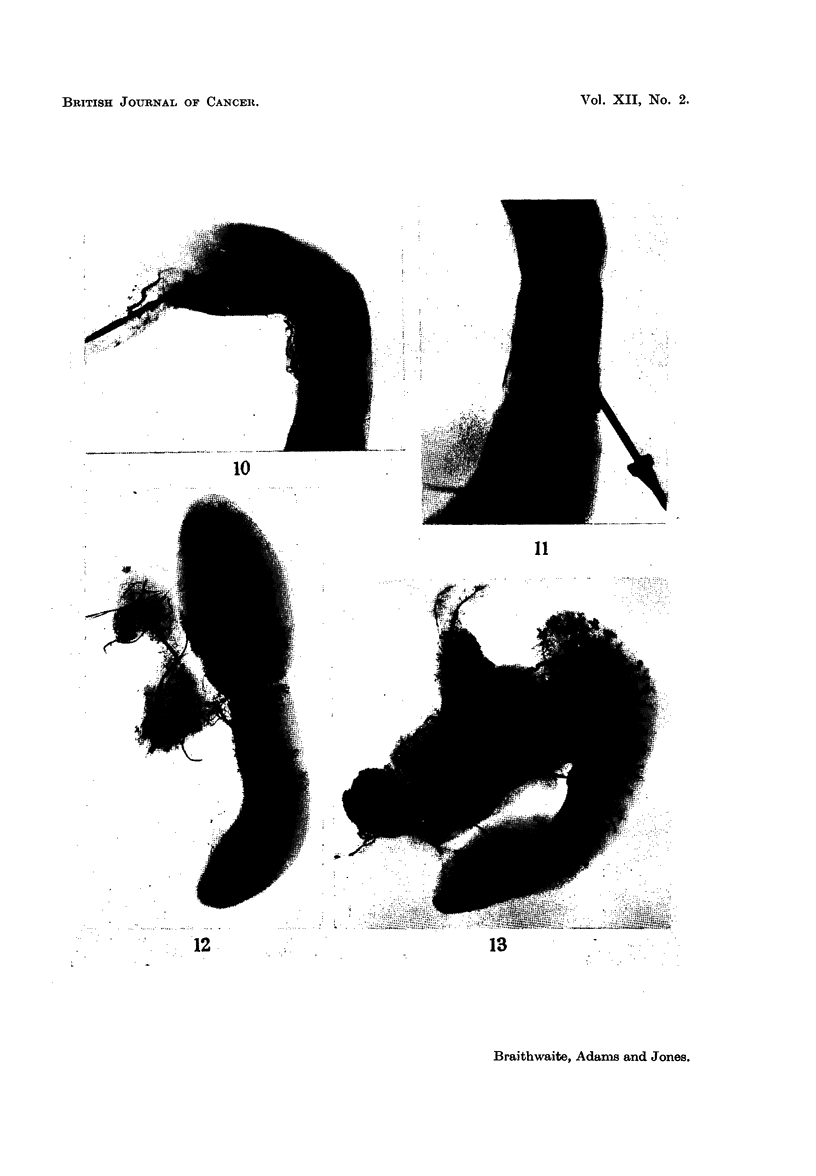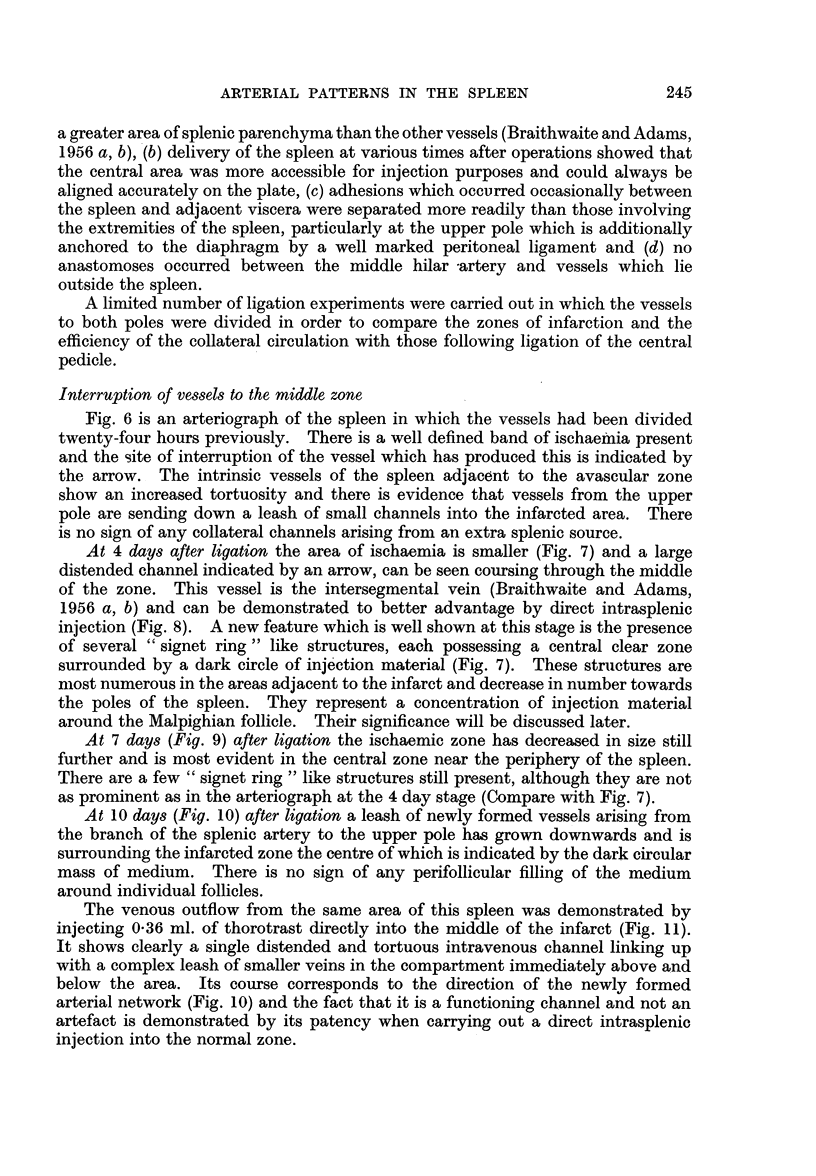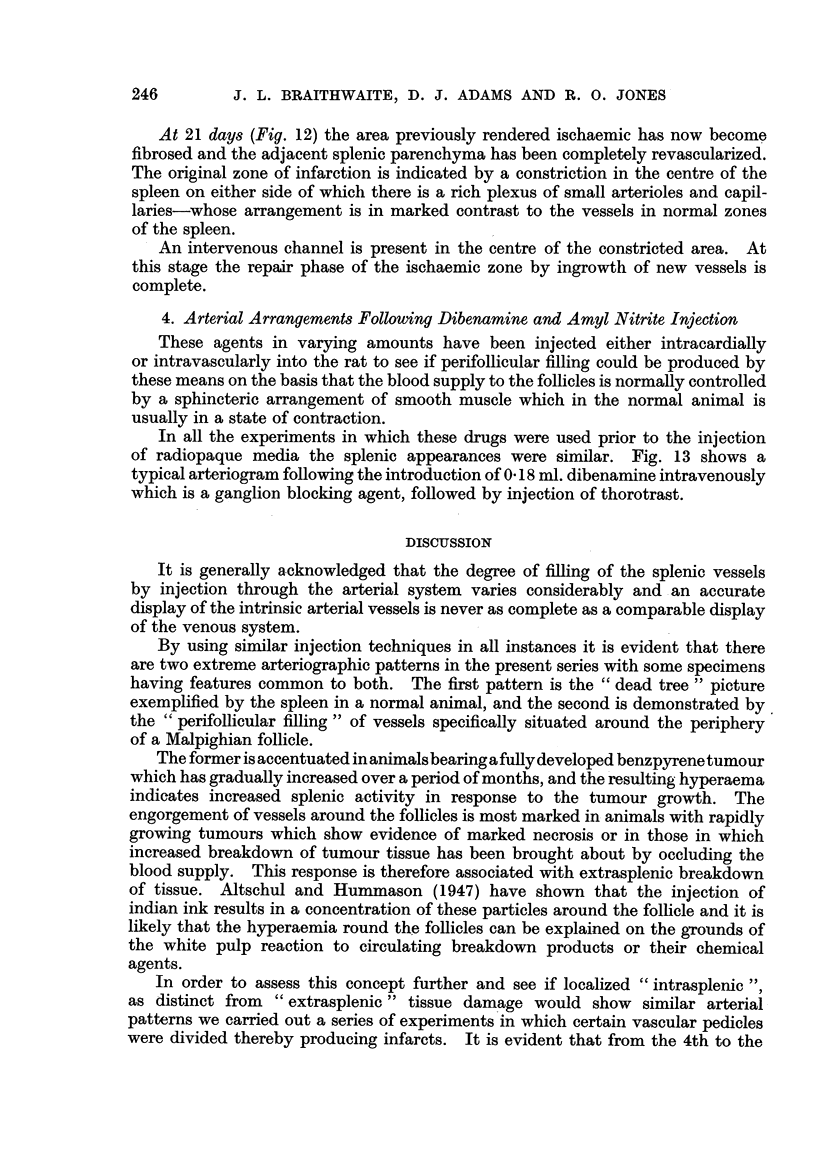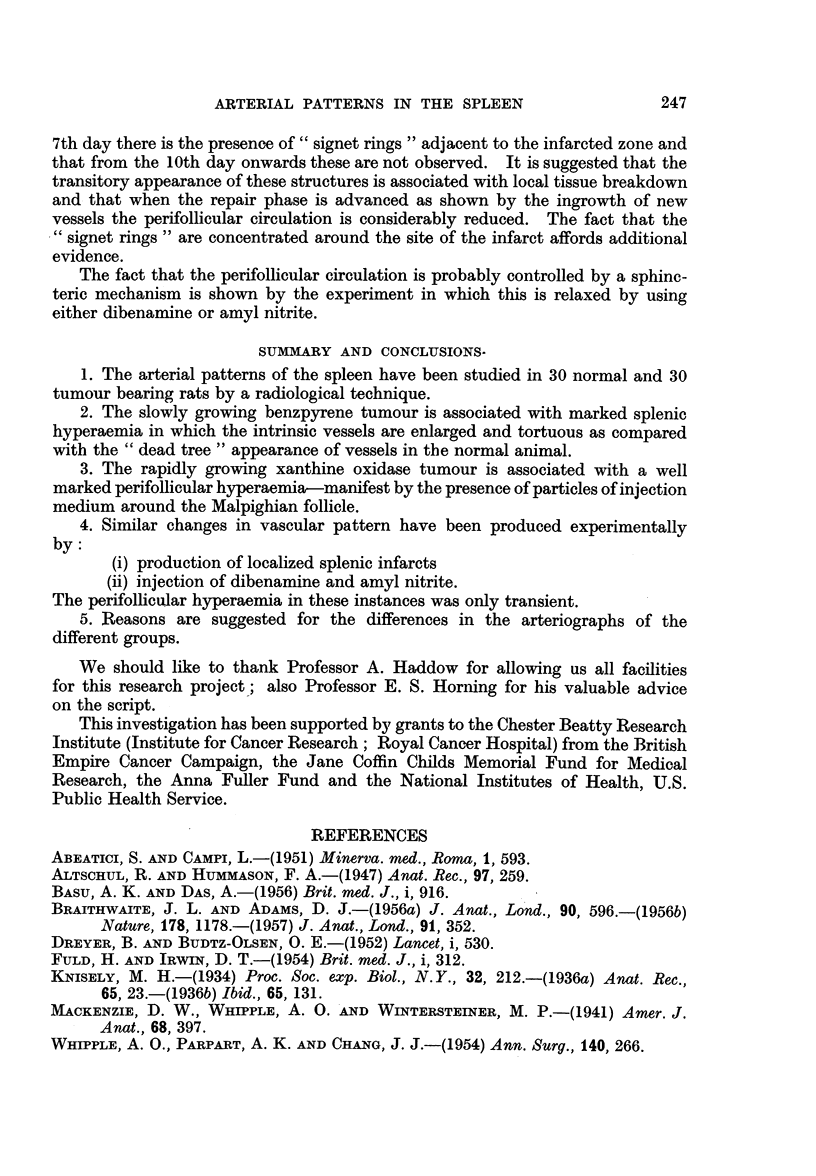# Arterial Patterns in the Spleen of Normal and Tumour Bearing Animals

**DOI:** 10.1038/bjc.1958.29

**Published:** 1958-06

**Authors:** J. L. Braithwaite, D. J. Adams, R. O. Jones

## Abstract

**Images:**


### ARTERIAL PATTERNS IN THE SPLEEN OF NORMAL AND TUMOUR BEARING ANIMALS

J. L. BRAITHWAITE, D. J. ADAMS AND R. O. JONES

*From the Department of Anatomy, University of Liverpool and the Chester Beatty Research Institute, Institute of Cancer Research, The Royal Cancer Hospital, London, S.W.3*

Received for publication March 14, 1958

THIS paper is concerned with the study of arterial patterns of the spleen in the normal animal (rat) and comparison between these findings and those present in experimental animals bearing certain tumours (benzpyrene induced and the xanthine tumour). It was stimulated by certain incidental findings in splenic vascularization when one of us (J. L. B.) was carrying out a study of the blood supply of experimental tumours and the effects of its occlusion, during an investigation at the Chester Beatty Research Institute.

An extensive literature already exists on the vascular arrangements of the spleen in the normal experimental animal and the contributions of Knisely (1934, 1936*a*, *b*) Mackenzie, Whipple and Wintersteiner (1941) and Whipple, Parpart and Chang (1954) using the transillumination technique are the most notable (on the intrinsic circulation) though their conclusions differ in certain respects. The possible value of using radiopaque injection material for the elucidation of splenic enlargement and the site of obstruction in the portal system of veins was first demonstrated by Abeatici and Campi (1951) in the dog. Several workers have since used a modified technique as a diagnostic procedure for portal hypertension in man, in which venous tributaries of the portal system and particularly the tributaries of the splenic veins show up clearly, notably Dreyer and Budtz-Olsen (1952), Fuld and Irwin (1954), Basu and Das (1956) and others.

We adopted a method similar to that of Abeatici and Campi (1951) in our earlier studies to clarify many features of the intrinsic venous drainage of the rat spleen. We had the advantage over previous workers that by using an experimental animal we could take several radiographs of each specimen, whereas the number of radiographs taken in man must of necessity be limited. The results of our previous investigations on the venous drainage of the rat spleen and the effects of its partial occlusion have already been published, Braithwaite and Adams (1956*a*, 1956*b*, 1957).

The present study on the arterial arrangements in normal and tumour bearing animals, employing similar injection techniques to those described in earlier papers presents a more striking contrast between the arterial patterns of the normal and pathological spleen than was the case following splenic venography.

### MATERIAL AND METHODS

The splenic arterial arrangements of normal rats were investigated in 30 instances; their weights ranged between 180 and 260 g. Similar investigations were conducted on the spleens of 30 tumour bearing rats (22 benzpyrene and 8 xanthine). In some of the tumour bearing animals the blood supply was occluded prior to sacrifice of the animal either by ligating or clamping the main vessels of supply to the tumour and noting any additional effect of these procedures on splenic vascularization.

The technique employed in displaying the arterial system of the spleen was the injection of a radiopaque medium (either micropaque, thorotrast or diaginol were used) into either the abdominal aorta, the splenic artery or one of its hilar branches in the living animal or in the animal recently killed by coal gas (to prevent intravascular clotting). Radiographs on Kodaline film were taken during the course of the injection and at various times after this with the spleen already in position on the plate.

In order to assess the efficiency of the collateral circulation and to try and produce experimentally similar arteriographic appearances to those encountered in the spleen of some of the tumour bearing animals certain additional procedures were employed in two groups of rats. In one infarction of a splenic zone was produced by ligation of its vessels of supply and the animals were sacrificed at varying periods after operation varying from 24 hours to two weeks and then injected and radiographed in a manner similar to that already described. In the second group certain agents such as dibenamine (ganglion blocking agent) and amyl nitrite (relaxant of smooth muscle) were injected intravascularly in graduated dosage prior to injection of radiopaque media.

### FINDINGS

### 1. *Normal Arterial Arrangements*

The splenic artery, a branch of the coeliac artery accompanied by the splenic vein approaches the spleen and gives off 5 to 7 hilar arteries which enter the organ at fairly regular intervals together with the corresponding hilar veins. Anastomoses between branches of the splenic artery and extrasplenic vessels occur at the upper and lower poles of the spleen, particularly between the hilar artery of the upper pole and the fundic arteries and between the branch to the lower pole and gastroepiploic vessels.

The main direction of the intrinsic arteries is at right angles to the long axis of the spleen and they break up within the organ into a tree-like arborization of smaller channels. It is evident that there are no anastomoses of macroscopic size between the arteries of adjacent zones (Fig. 1).

### 2. *Arterial Arrangements in the Tumour Bearing Animals*

In the rat the vascularization of two types of tumour was studied particularly—one was the tumour induced by insertion of a benzpyrene tablet and the other followed the introduction of tumour tissue originally induced by xanthine oxidase. The former is comparatively slow in its growth and takes about 9–10 months to become fully established; the latter grows very rapidly and reaches a considerable size between 6 and 10 days after implantation.

The typical arterial arrangements of the spleen in an animal with an advanced benzpyrene tumour are shown in Fig. 2; comparing this arteriograph with the arrangements in the normal animal (Fig. 1) it will be noted that the former presents a marked increase in vascularity and the injection mass has penetrated into the finest ramifications of the intrinsic vessels filling the splenic parenchyma throughout. It is not possible to demarcate the territorial supply of each hilar-vessel (of which there are seven in number in this instance) as is the case with the normal (Fig. 1). Further the larger intrasplenic vessels show an increased tortuosity and this dilatation is also reflected in the extrasplenic course of these hilar vessels. This marked splenic hyperaemia in the animal bearing the benzpyrene tumour is a manifestation of increased splenic activity and is a direct response to the growing tumour.

The arteriographs in the rapidly growing xanthine tumour which show advanced necrotic changes within the tumour differ from those in the previous series. The typical splenic appearance is emphasized if the rate of breakdown of the tumour is increased by cutting off its blood supply. Fig. 3 is an arteriograph of an animal bearing a xanthine oxidase tumour in the right thigh. In this animal the blood supply was occluded on the sixth day after implantation of the tumour tissue, and six days after the occlusion of the vessels, the animal was sacrificed. The site of interruption is clearly seen along the anterior division of the femoral artery. The spleen is full of opacities with a cotton wool appearance around the edges though being in the main circular in shape and corresponding to vascular engorgement in the region of the Malpighian follicles.

Similar appearances have also been noted in the spleens of mice bearing mammary carcinomata following interruption of their blood supply either by vessel ligation or by clamping the main vascular pedicles to the tumour. Fig. 4 and 5 show varying degrees of splenic opacity following these procedures. In Fig. 4 the spleen is completely filled and in Fig. 5 the opacities are scattered irregularly throughout the parenchyma (the lower pole of the spleen being indicated by the arrow). All illustrate an engorgement of vessels around the periphery of the Malpighian follicle.

### 3. *Arterial Arrangements Following Interruption of Hilar Vessels*

The majority of ligation experiments carried out in this series involved interruption of the hilar vessels supplying the central zone of the spleen for the following reasons, (*a*) these vessels were generally larger than the remainder and supplied

---

EXPLANATION OF PLATES

FIG. 1.—Arteriograph of the spleen in the normal rat. × 2·2.
FIG. 2.—Arteriograph of the spleen in an animal with an advanced benzpyrene tumour in the right thigh. × 1·5.
FIG. 3.—Arteriograph of an animal with a xanthine oxidase tumour in the right thigh. Note the splenic opacities.
FIG. 4.—Arteriograph of a mouse with a mammary carcinoma showing multiple splenic opacities throughout the spleen.
FIG. 5.—Arteriograph of a mouse with mammary carcinomata showing splenic opacities scattered irregularly throughout the spleen.
FIG. 6.—Arteriograph of spleen 24 hours after the production of an infarct.
FIG. 7.—Arteriograph of spleen 4 days after the production of an infarct.
FIG. 8.—Venograph of spleen, following direct intrasplenic injection into the infarcted area 4 days after the production of an infarct.
FIG. 9.—Arteriograph of spleen 7 days after the production of an infarct.
FIG. 10.—Arteriograph of spleen 10 days after the production of an infarct. × 2·5.
FIG. 11.—Venograph of spleen following a direct intrasplenic injection into the infarcted area 10 days after operation. × 2·5.
FIG. 12.—Arteriograph of spleen 21 days after the production of an infarct. × 2.
FIG. 13.—Arteriograph of spleen from an animal previously injected with 0·18 ml. of dibenamine. × 2.

1

2

3

4

Braithwaite, Adams and Jones.

5

6

7

8

9

Braithwaite, Adams and Jones,

10

11

12

13

Braithwaite, Adams and Jones.

a greater area of splenic parenchyma than the other vessels (Braithwaite and Adams, 1956 *a*, *b*), (*b*) delivery of the spleen at various times after operations showed that the central area was more accessible for injection purposes and could always be aligned accurately on the plate, (*c*) adhesions which occurred occasionally between the spleen and adjacent viscera were separated more readily than those involving the extremities of the spleen, particularly at the upper pole which is additionally anchored to the diaphragm by a well marked peritoneal ligament and (*d*) no anastomoses occurred between the middle hilar artery and vessels which lie outside the spleen.

A limited number of ligation experiments were carried out in which the vessels to both poles were divided in order to compare the zones of infarction and the efficiency of the collateral circulation with those following ligation of the central pedicle.

*Interruption of vessels to the middle zone*

Fig. 6 is an arteriograph of the spleen in which the vessels had been divided twenty-four hours previously. There is a well defined band of ischaemia present and the site of interruption of the vessel which has produced this is indicated by the arrow. The intrinsic vessels of the spleen adjacent to the avascular zone show an increased tortuosity and there is evidence that vessels from the upper pole are sending down a leash of small channels into the infarcted area. There is no sign of any collateral channels arising from an extra splenic source.

*At 4 days after ligation* the area of ischaemia is smaller (Fig. 7) and a large distended channel indicated by an arrow, can be seen coursing through the middle of the zone. This vessel is the intersegmental vein (Braithwaite and Adams, 1956 *a*, *b*) and can be demonstrated to better advantage by direct intrasplenic injection (Fig. 8). A new feature which is well shown at this stage is the presence of several " signet ring " like structures, each possessing a central clear zone surrounded by a dark circle of injection material (Fig. 7). These structures are most numerous in the areas adjacent to the infarct and decrease in number towards the poles of the spleen. They represent a concentration of injection material around the Malpighian follicle. Their significance will be discussed later.

*At 7 days (Fig. 9) after ligation* the ischaemic zone has decreased in size still further and is most evident in the central zone near the periphery of the spleen. There are a few " signet ring " like structures still present, although they are not as prominent as in the arteriograph at the 4 day stage (Compare with Fig. 7).

*At 10 days (Fig. 10) after ligation* a leash of newly formed vessels arising from the branch of the splenic artery to the upper pole has grown downwards and is surrounding the infarcted zone the centre of which is indicated by the dark circular mass of medium. There is no sign of any perifollicular filling of the medium around individual follicles.

The venous outflow from the same area of this spleen was demonstrated by injecting 0·36 ml. of thorotrast directly into the middle of the infarct (Fig. 11). It shows clearly a single distended and tortuous intravenous channel linking up with a complex leash of smaller veins in the compartment immediately above and below the area. Its course corresponds to the direction of the newly formed arterial network (Fig. 10) and the fact that it is a functioning channel and not an artefact is demonstrated by its patency when carrying out a direct intrasplenic injection into the normal zone.

*At* 21 *days* (*Fig.* 12) the area previously rendered ischaemic has now become fibrosed and the adjacent splenic parenchyma has been completely revascularized. The original zone of infarction is indicated by a constriction in the centre of the spleen on either side of which there is a rich plexus of small arterioles and capillaries—whose arrangement is in marked contrast to the vessels in normal zones of the spleen.

An intervenous channel is present in the centre of the constricted area. At this stage the repair phase of the ischaemic zone by ingrowth of new vessels is complete.

4. *Arterial Arrangements Following Dibenamine and Amyl Nitrite Injection*

These agents in varying amounts have been injected either intracardially or intravascularly into the rat to see if perifollicular filling could be produced by these means on the basis that the blood supply to the follicles is normally controlled by a sphincteric arrangement of smooth muscle which in the normal animal is usually in a state of contraction.

In all the experiments in which these drugs were used prior to the injection of radiopaque media the splenic appearances were similar. Fig. 13 shows a typical arteriogram following the introduction of 0·18 ml. dibenamine intravenously which is a ganglion blocking agent, followed by injection of thorotrast.

### DISCUSSION

It is generally acknowledged that the degree of filling of the splenic vessels by injection through the arterial system varies considerably and an accurate display of the intrinsic arterial vessels is never as complete as a comparable display of the venous system.

By using similar injection techniques in all instances it is evident that there are two extreme arteriographic patterns in the present series with some specimens having features common to both. The first pattern is the " dead tree " picture exemplified by the spleen in a normal animal, and the second is demonstrated by the " perifollicular filling " of vessels specifically situated around the periphery of a Malpighian follicle.

The former is accentuated in animals bearing a fully developed benzpyrene tumour which has gradually increased over a period of months, and the resulting hyperaema indicates increased splenic activity in response to the tumour growth. The engorgement of vessels around the follicles is most marked in animals with rapidly growing tumours which show evidence of marked necrosis or in those in which increased breakdown of tumour tissue has been brought about by occluding the blood supply. This response is therefore associated with extrasplenic breakdown of tissue. Altschul and Hummason (1947) have shown that the injection of indian ink results in a concentration of these particles around the follicle and it is likely that the hyperaemia round the follicles can be explained on the grounds of the white pulp reaction to circulating breakdown products or their chemical agents.

In order to assess this concept further and see if localized " intrasplenic ", as distinct from " extrasplenic " tissue damage would show similar arterial patterns we carried out a series of experiments in which certain vascular pedicles were divided thereby producing infarcts. It is evident that from the 4th to the

7th day there is the presence of " signet rings " adjacent to the infarcted zone and that from the 10th day onwards these are not observed. It is suggested that the transitory appearance of these structures is associated with local tissue breakdown and that when the repair phase is advanced as shown by the ingrowth of new vessels the perifollicular circulation is considerably reduced. The fact that the " signet rings " are concentrated around the site of the infarct affords additional evidence.

The fact that the perifollicular circulation is probably controlled by a sphincteric mechanism is shown by the experiment in which this is relaxed by using either dibenamine or amyl nitrite.

### SUMMARY AND CONCLUSIONS

1. The arterial patterns of the spleen have been studied in 30 normal and 30 tumour bearing rats by a radiological technique.
2. The slowly growing benzpyrene tumour is associated with marked splenic hyperaemia in which the intrinsic vessels are enlarged and tortuous as compared with the " dead tree " appearance of vessels in the normal animal.
3. The rapidly growing xanthine oxidase tumour is associated with a well marked perifollicular hyperaemia—manifest by the presence of particles of injection medium around the Malpighian follicle.
4. Similar changes in vascular pattern have been produced experimentally by :
    (i) production of localized splenic infarcts
    (ii) injection of dibenamine and amyl nitrite.

The perifollicular hyperaemia in these instances was only transient.

5. Reasons are suggested for the differences in the arteriographs of the different groups.

We should like to thank Professor A. Haddow for allowing us all facilities for this research project; also Professor E. S. Horning for his valuable advice on the script.

This investigation has been supported by grants to the Chester Beatty Research Institute (Institute for Cancer Research ; Royal Cancer Hospital) from the British Empire Cancer Campaign, the Jane Coffin Childs Memorial Fund for Medical Research, the Anna Fuller Fund and the National Institutes of Health, U.S. Public Health Service.

### REFERENCES

ABEATICI, S. AND CAMPI, L.—(1951) *Minerva. med., Roma*, **1**, 593.

ALTSCHUL, R. AND HUMMASON, F. A.—(1947) *Anat. Rec.*, **97**, 259.

BASU, A. K. AND DAS, A.—(1956) *Brit. med. J.*, i, 916.

BRAITHWAITE, J. L. AND ADAMS, D. J.—(1956*a*) *J. Anat., Lond.*, **90**, 596.—(1956*b*) *Nature*, **178**, 1178.—(1957) *J. Anat., Lond.*, **91**, 352.

DREYER, B. AND BUDTZ-OLSEN, O. E.—(1952) *Lancet*, i, 530.

FULD, H. AND IRWIN, D. T.—(1954) *Brit. med. J.*, i, 312.

KNISELY, M. H.—(1934) *Proc. Soc. exp. Biol., N.Y.*, **32**, 212.—(1936*a*) *Anat. Rec.*, **65**, 23.—(1936*b*) *Ibid.*, **65**, 131.

MACKENZIE, D. W., WHIPPLE, A. O. AND WINTERSTEINER, M. P.—(1941) *Amer. J. Anat.*, **68**, 397.

WHIPPLE, A. O., PARPART, A. K. AND CHANG, J. J.—(1954) *Ann. Surg.*, **140**, 266.